# Parental Knowledge and Practices Related to Foreign Body Aspiration in Children in Makkah, Saudi Arabia

**DOI:** 10.7759/cureus.34816

**Published:** 2023-02-09

**Authors:** Bassam M Bin Laswad, Hawazen M Alsulaimani, Mohanned M Alomairi, Rola R Alsulami, Sultan F Alobaidi, Hazem Aljabri, Shahad T Alsaidi, Mohammed H Ageel

**Affiliations:** 1 Department of Medicine and Surgery, College of Medicine, Umm Al-Qura University, Makkah, SAU; 2 Department of Surgery, College of Medicine, Umm Al-Qura University, Makkah, SAU

**Keywords:** life-threatening emergency, choking hazards, parental awareness, education, parental knowledge, foreign body aspiration, inhalation, mortality, rigid bronchoscopy, aerodigestive tract

## Abstract

Background

Foreign body aspiration (FBA) is a life-threatening event and one of the most common causes of mortality in children. As it has different clinical presentations, parental knowledge is essential for early management to prevent complications.

Objectives

This study was designed to assess the knowledge and practices relating to FBA in children among parents living in Makkah city, Saudi Arabia.

Methods

An online questionnaire was designed using Google Forms (Google LLC, Mountain View, California, United States) and distributed in October 2022 among parents living in Makkah city. After data collection, an appropriate statistical analysis was conducted.

Results

A total of 1087 parents enrolled in this study; 63.9% were women and the majority were married 93%. Additionally, 52% of the parents had at least three children. Moreover, 17.6% had an experience of a child having aspirated a foreign body once. The Internet was the most popular source of information on FBA (43.5%). Furthermore, the parents had poor levels of knowledge and practices related to FBA (65.4% and 78.6%, respectively).

Conclusion

This study reported that parental levels of knowledge of FBA and FBA practices were inadequate. There is a need to increase awareness, which will lead to better outcomes.

## Introduction

Foreign body aspiration (FBA) is a serious condition that can result in death, in addition to its significant association with social and economic outcomes [1]. This condition occurs more often in children below the age of five years [2]. FBA has a variable clinical presentation ranging from being asymptomatic to severe respiratory distress and death [3]. Parents witnessing and reporting the incident of inhalation to a physician is helpful for early diagnosis and intervention [4,5]. Furthermore, the late diagnosis of FBA can be associated with a high rate of mortality and morbidity [6]. In fact, FBA has been reported to cause mortality in 7% of children less than or equal to three years of age [7-9].

Therefore, parental awareness of the signs and symptoms of FBA as well as the practice of its first aid is crucial for preventing late presentation to care and thus lowering morbidity and fatality rates [4]. Previous studies on this topic have been conducted in Saudi Arabia and other nations, including a study conducted in the Al Qassim region of Saudi Arabia that assessed parental knowledge and practices with regard to FBA in children [3,4,10,11]. However, no study on this has thus far been conducted in Makkah city. Hence, the primary aim of our study is to evaluate parental knowledge and FBA practices in Makkah, Saudi Arabia.

## Materials and methods

Study design and participants

This cross-sectional study was carried out in October 2022 on the general population of Makkah, Saudi Arabia. We enrolled both male and female parents, both Saudi and non-Saudi, living in Makkah city. Participants had to be over 18 years and agree to participate.

Ethical considerations and sample size

A self-administered survey through Google Forms (Google LLC, Mountain View, California, United States) was used to collect the data. This was distributed via social media platforms after obtaining ethical approval from the Biomedical Ethics Committee at the College of Medicine, Umm Al-Qura University, Makkah, Saudi Arabia (Approval number: HAPO-02-K-012-2022-09-1218).

We used the OpenEpi (version 3.01) website to estimate the sample size, keeping the confidence interval at 95% [12]. This estimated a minimum sample size of 385 participants. However, we collected 1087 responses to enhance the generalizability of the results and guarantee their accuracy.

Study tool

We used a validated assessment tool employed in a previous study [4]. The questionnaire was translated into Arabic. In order to assess the questionnaire’s readability and simplicity, a specialist was selected to serve the purpose. Furthermore, a pilot study was carried out in which we asked 30 individuals to complete the questionnaire, then essential minor changes were done. The data from the pilot study were excluded from the final dataset used in the study.

The survey included three sections. The first section collected the participants’ demographic data, parental knowledge of FBA was assessed in the second section, and their FBA practices were assessed in the third section.

Scoring

Eight questions were used to assess parental knowledge of FBA. For every correct (incorrect) answer, the respondent scored 1 (0). The scores of all eight questions were added to calculate the total parental knowledge score. We classified parents as having poor (score of 1-5) or good (score of 6-8) knowledge using the mean score as the cutoff point. Moreover, parental FBA practices were also assessed using eight questions and a score ranging from 7-20 was generated. The questions were divided into two types. The first four questions were answered using a five-point Likert scale (1 for “always”, 2 for “often”, 3 for “sometimes”, 4 for “rarely”, and 5 for “never”). To avoid bias in the score, we reverse-coded the negative questions. For the second four questions, for every correct (incorrect) answer, the participant scored 1 (0). The total score was obtained by adding the scores of all eight questions. The participants were classified as having good FBA practices if they scored 13-20 points and poor FBA practices if they scored 7-12 points.

Statistical analysis

Data were analyzed using IBM SPSS Statistics for Windows, Version 26.0 (Released 2019; IBM Corp., Armonk, New York, United States). To assess the relationships among the variables, the qualitative data were expressed as numbers and percentages, and the Chi-squared test (χ2) was used. The quantitative data were expressed as mean and standard deviation (Mean ± SD), and the non-parametric variables were tested using the Mann-Whitney test. A p-value < 0.05 was considered to be statistically significant.

## Results

A total of 1087 parents were involved in the present study. The mean age of the studied parents was 40.36 ± 9.76 years; 63.9% were women and 93% were married. Of them, 59.1% had a bachelor’s degree and 15% were healthcare workers. Further, 52% had more than three children and the youngest child was one to five years for 38.3%. Of the participants, 17.6% and 2.8% had experience of a child aspirating foreign bodies once and twice, respectively, while 2.7% had experience of more than two such events. More than half of the participants (60.8%) had heard or read about FBA (Table 1).

**Table 1:** Distribution of studied parents according to their demographic characters and children's data (n= 1087) Data are presented as numbers and percentages (%) except for age which is presented as mean and standard deviation (Mean ± SD).

Variable	Total
Age in years	40.36 ±9.76
Gender	
Female	695 (63.9%)
Male	392 (36.1%)
Marital status	
Widow	20 (1.8%)
Married	1011 (93%)
Divorced	56 (5.2%)
Education	
Less than secondary school	72 (6.6%)
Secondary school	190 (17.5%)
Diploma	90 (8.3%)
Bachelor	642 (59.1%)
Postgraduate	93 (8.6%)
Healthcare worker	
No	924 (85%)
Yes	163 (15%)
Children number	
1-3	522 (48%)
>3	565 (52%)
Age of youngest child	
<1 year	145 (13.3%)
1-5 years	416 (38.3%)
6-10 years	265 (24.4%)
>10 years	261 (24%)
Have any of your children aspirated a foreign body?	
No	837 (77%)
Once	191 (17.6%)
Twice	30 (2.8%)
More than twice	29 (2.7%)
Have you heard or read about foreign body aspiration?	
No	426 (39.2%)
Yes	661 (60.8%)

**Figure 1:**
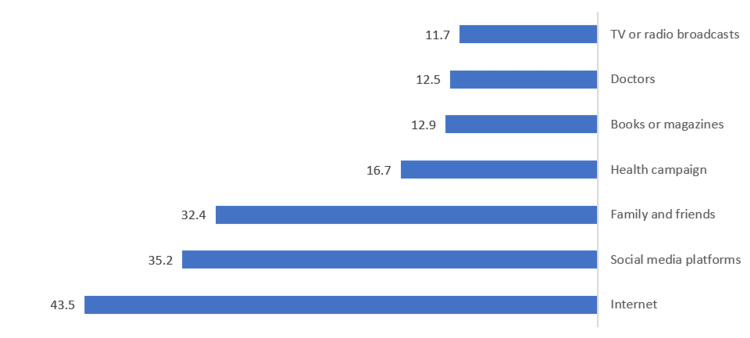
Percentage distribution of sources of information about FBA (n=1087) FBA: foreign body aspiration

The most common sources of information about FBA were the Internet (43.5%), social media platforms (35.2%), and family and friends (32.4%) (Figure 1).

Altogether, 65.8% correctly knew that children aged one to five years are at the highest risk of aspirating foreign bodies. About 68.9% agreed that children should not be offered peanuts until they reach four years and 71.8% disagreed that only items with a smooth surface can be aspirated. About 46.2% disagreed that the absence of choking is an assuring sign that the item has been dislodged and 67.3% disagreed that if the foreign body causes no symptoms, it is okay to delay removal. The majority (90.4%) agreed that talking while chewing may lead to aspiration. Only 24.1% disagreed that X-rays can detect all foreign bodies. Most of the parents (80.1%) correctly knew that children are at risk of aspirating both organic items such as nuts and non-organic items such as small plastic toys (Table 2).

**Table 2:** Responses of the participants to knowledge items regarding foreign body aspiration (n=1087) *Signifies correct answer

Statement	n (%)
At what age children are at the highest risk to aspirate foreign bodies	
<1 year	284 (26.1)
1-5 years*	715 (65.8)
6-10 years	60 (5.5)
>10 years	28 (2.6)
Children shouldn’t be offered peanuts until they are 4 years old	
Agree*	749 (68.9)
Disagree	338 (31.1)
Only items with a smooth surface can be aspirated	
Agree	307 (28.2)
Disagree *	780 (71.8)
Absence of choking is an assuring sign that the item has gone away	
Agree	585 (53.8)
Disagree*	502 (46.2)
If the foreign body causes no symptoms, it is okay to delay removal	
Agree	355 (32.7)
Disagree*	732 (67.3)
Talking while chewing may lead to aspiration	
Agree *	983 (90.4)
Disagree	104 (9.6)
X-rays can detect all foreign bodies	
Agree	825 (75.9)
Disagree*	262 (24.1)
Which of the following items are children at risk to aspirate?	
Organic like nuts	32 (2.9)
Non-organic like small plastic toys	148 (13.6)
Both*	871 (80.1)
None	36 (3.3)

Table 3 shows that 23.3% of the parents never buy toys with small parts that can be aspirated, while 31.1% never let their child play without supervision. The majority (66.1%) keep small items out of the reach of children and 34.4% never let their child eat without supervision. Only 20.6% would not attempt to remove the foreign body inside the child’s mouth with their fingers. Moreover, 68.4% would attempt back slaps or abdominal thrusts if the child was choking and able to talk. Most of the participants (92.7%) would attempt back slaps or abdominal thrust if the child was choking and unable to talk. Only 17.4% would not go to hospital if there were no symptoms or if the symptoms had subsided shortly after (Table 3).

**Table 3:** Responses of the participants to practice items regarding foreign body aspiration (n=1087) ^#^Indicates reverse answer; *Signifies correct answer

Variable	n (%)
Do you buy toys with small parts that can be aspirated?	
Always	42 (3.9)
Often	109 (10)
Sometimes	429 (39.5)
Rarely	254 (23.4)
Never*	253 (23.3)
Do let your child play without supervision?	
Always	18 (1.7)
Often	72 (6.6)
Sometimes	307 (28.2)
Rarely	352 (32.4)
Never*	338 (31.1)
Do you keep small items out of the reach of children?^ #^	
Always*	719 (66.1)
Often	198 (18.2)
Sometimes	116 (10.7)
Rarely	37 (3.4)
Never	17 (1.6)
Do let your child eat without supervision?	
Always	14 (1.3)
Often	47 (4.3)
Sometimes	234 (21.5)
Rarely	418 (38.5)
Never*	374 (34.4)
Would you attempt to remove the foreign body inside the child’s mouth with your fingers?	
No*	224 (20.6)
Yes	863 (79.4)
Would you attempt to do back slaps or abdominal thrusts if the child is choking and able to talk?	
No	343 (31.6)
Yes*	477 (68.4)
Would you attempt to do back slaps or abdominal thrusts if the child is choking and not able to talk?	
No*	79 (7.3)
Yes	1008 (92.7)
Would you go to a hospital if there are no symptoms or if the symptoms subsided shortly after?	
No*	189 (17.4)
Yes	898 (82.6)

**Figure 2:**
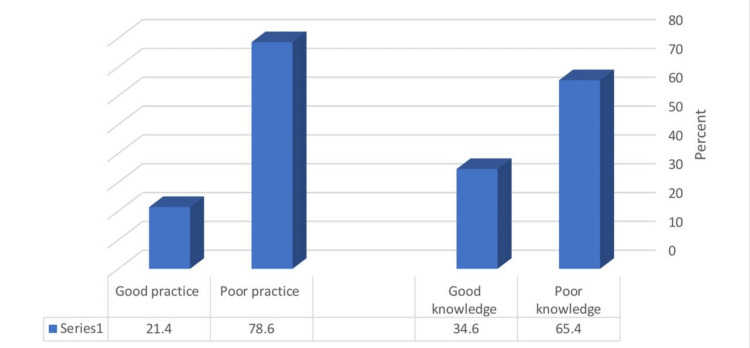
Percentage distribution of parents according to their knowledge and practice levels (n=1087)

The mean knowledge and practice scores were 5.75 ± 1.55 and 9.57 ± 1.2, respectively. Of the studied parents, 65.4% and 34.6% had poor and good levels of knowledge of FBA, respectively, whereas 78.6% and 21.4% had poor and good FBA practices, respectively (Figure 2).

The association between the knowledge of FBA and FBA practices of the participants and their demographic characteristics is shown in Table 4 and Table 5. It was found that women and those with a bachelor's degree had a significantly higher likelihood of having good knowledge of FBA (p ≤ 0.05) (Table 4). Furthermore, parents with a bachelor's degree had a significantly higher likelihood of having good FBA practices (p ≤ 0.05) (Table 5).

**Table 4:** Relationship between knowledge level about FBA and participants' demographic characters and children's data (n=1087) Data are presented as numbers and percentages (%) except for age which is presented as mean and standard deviation (Mean ± SD) FBA: foreign body aspiration

Variable	Knowledge level		χ2	p-value
	Poor, n (%)	Good, n (%)		
Age in years	40.74 ± 9.86	39.64 ± 9.56	1.52	0.127
Gender			6.09	0.014
Female	436 (61.3)	259 (68.9)		
Male	275 (38.7)	117 (31.1)		
Marital status			1.79	0.409
Widow	13 (1.8)	7 (1.9)		
Married	666 (93.7)	345 (91.8)		
Divorced	32 (4.5)	24 (6.4)		
Education			18.11	0.001
Less than secondary school	59 (8.3)	13 (3.5)		
Secondary school	135 (19)	55 (14.6)		
Diploma	55 (7.7)	35 (9.3)		
Bachelor	412 (57.9)	230 (61.2)		
Postgraduate	50 (7)	43 (11.4)		
Health care worker			2.95	0.086
No	614 (86.4)	310 (82.4)		
Yes	97 (13.6)	66 (17.6)		
Number of children			0.67	0.411
1-3	335 (47.1)	187 (49.7)		
>3	376 (52.9)	189 (50.3)		
Age of youngest child			1.05	0.787
<1 year	91 (12.8)	54 (14.4)		
1-5 years	277 (39)	139 (37)		
6-10 years	176 (24.8)	89 (23.7)		
>10 years	167 (23.5)	94 (25)		

**Table 5:** Relationship between practice level and participants' demographic characters and children's data (n=1087) Data are presented as numbers and percentages (%) except for age which is presented as mean and standard deviation (Mean ± SD)

Variable	Practice level		χ2	p-value
	Poor, n (%)	Good, n (%)		
Age in years	40.2 ± 9.86	40.95 ± 9.4	1	0.313
Gender			1.93	0.165
Female	537 (62.9)	158 (67.8)		
Male	317 (37.1)	75 (32.2)		
Marital status			3.14	0.208
Widow	13 (1.5)	7 (3)		
Married	794 (93)	217 (93.1)		
Divorced	47 (5.5)	9 (3.9)		
Education			13.9	0.008
Less than secondary school	54 (6.3)	18 (7.7)		
Secondary school	137 (16)	53 (22.7)		
Diploma	63 (7.4)	27 (11.6)		
Bachelor	527 (61.7)	115 (49.4)		
Postgraduate	73 (8.5)	20 (8.6)		
Health care worker			1.57	0.21
No	732 (85.7)	192 (82.4)		
Yes	122 (14.3)	41 (17.6)		
Number of children			0.76	0.383
1-3	416 (48.7)	106 (45.5)		
>3	438 (51.3)	127 (54.5)		
Age of youngest child			1.09	0.778
<1 year	115 (13.5)	30 (12.9)		
1-5 years	330 (38.6)	86 (36.9)		
6-10 years	210 (24.6)	55 (23.6)		
>10 years	199 (23.3)	62 (26.6)		

## Discussion

FBA is a respiratory emergency in young children, especially those under the age of five, that needs urgent care and management, as it is considered to be the leading cause of morbidity and mortality among children [3,13]. Furthermore, 44% of infants and 7% of preschool children die as a result of FBA [2,14]. Hence, assessing parents’ knowledge of FBA and FBA practices could help substantially lower the mortality rate. This study investigates parental knowledge of FBA and FBA practices.

Among the 1087 parents surveyed, our results showed a poor level of knowledge and low level of FBA practices (65.4% and 78.6%, respectively), which agrees with a similar study conducted in Al Qassim region [4] Their participants had poor knowledge of FBA (63.1%) and poor FBA practices (55.3%), whereas another study carried in the eastern province of Saudi Arabia reported a high level of awareness (60.3%) about aerodigestive foreign bodies in pediatrics [15]. In comparison to the eastern province study, which aimed to evaluate parents’ awareness of the risk of aerodigestive foreign bodies in children, our study expanded the scope to assess the FBA practices and knowledge of FBA of parents.

In the present study, the mean age of the parents was 40.36 ± 9.76 years and 63.9% of them were women. These results are consistent with Al Qassim region study [4]. On the other hand, a Japanese study targeted mothers only and their mean age was 32 years [11]. Additionally, more than three-quarters of our participants’ children (77%) had not experienced FBA; however 17.6%, 2.8%, and 2.7% had experienced one, two, and more than two events, respectively, which agrees with the Al Qassim study [4]. In our study, 60.8% of the parents had heard or read about FBA compared with 41.3% of Al Qassim participants [4].

As parents’ knowledge plays an important role in lowering the incidence of FBA among children, we assessed their knowledge in our study. We found that most of the parents (68.9%) concurred that children ought not to be offered peanuts until they reach four years of age. In addition, the vast majority of them (80.1%) agreed that items such as nuts and plastic toys are a possible hazard to be aspirated among children. These findings are consistent with several international and local studies [4,11,16,17]. Yet, a local study in the outpatient clinics of two main hospitals in the eastern province of Saudi Arabia demonstrated that 80% of college-graduated parents did not recognize peanuts as a cause of FBA, while all the parents from the lower-education group knew that peanuts were a cause of FBA. Moreover, half of the college-graduated parents were not convinced that children below three years should not be given peanuts [3].

As for the FBA practices of parents, poor FBA practices of parents were also reported by a study conducted in Jordan [17]. According to its results, more than half of the mothers demonstrated poor practices. Furthermore, the Al Qassim study reported similar results, finding that only 44.7% of parents had good FBA practices, while 55.3% had poor FBA practices [4].

It’s important to mention that the parents’ sociodemographic characteristics directly influence their knowledge of FBA and FBA practices. We found that the female participants with bachelor's degrees had a significantly higher level of knowledge than the other participants (p ≤ 0.05). The participants with bachelor's degrees also had a higher level of FBA practices (p ≤ 0.05). Furthermore, the Al Qassim study documented that parents who have heard about FBA and who have fewer children demonstrate good knowledge and practices, in contrast to our findings [4].

Another factor that plays an important role in this subject's literacy is the participants' information source. According to the literature, there are disparities in reports on the source of information. The study by Singh et al. showed that friends and visual media dominate the information sources of FBA [18]. However, another study conducted in Jeddah, Saudi Arabia, reported that doctors are a reliable source of information [19]. In our study, the results demonstrated that the Internet (43.5%), social media platforms (35.2%), and family and friends (32.4%) were the most mentioned sources of information on parents’ knowledge of FBA and FBA practices. It is important to note that doctors and TV or radio broadcasts were much less chosen as sources of information (12.5% and 11.7%, respectively).

Based on these findings, to avoid the risk and reduce the incidence of FBA, it is important to educate the community and sensitize them to the risks and bad habits that might lead to this incident. The awareness of the community, especially parents, ought to be increased through health campaigns, for example. In light of our findings, which showed that the Internet was the most cited source of information, parents ought to be taught the correct method to search for and confirm the correct information online. Although the Internet is not always a reliable source, it provides fundamental knowledge. Family physicians and pediatricians also play an important role. Hence, they must help educate parents during routine visits to the clinic and through social media platforms, which can raise the awareness level and prevent serious sequels.

Limitations

Despite the large study sample, the current study still represented only a single region in Saudi Arabia. Hence, this might affect the generalizability of the results to other Saudi Arabian regions. Sampling bias is another possible limitation of this study. As female parents responded to the survey more than males, a balanced sample may enhance the generalizability of the findings.

## Conclusions

FBA in children is a pediatric emergency with a high mortality rate and serious consequences. This study showed insufficient levels of knowledge of FBA and FBA practices. Additionally, the need to raise awareness of FBA among parents was evident in this study, which can be accomplished through the help of family physicians and pediatricians by explaining to parents what to do and how to act in the case of an FBA event with their child; this would help in preventing complications and lower the mortality associated with FBA.
